# Supplementary material for: Rational Design, Synthesis and Preliminary Evaluation of Novel Fusarinine C-Based Chelators for Radiolabeling with Zirconium-89
Source: Biomolecules. 2019 Mar 6;9(3):91. doi: 10.3390/biom9030091 (PMC6468543; doi:10.3390/biom9030091)
Supplement: Supplementary file 1 [file biomolecules-09-00091-s001.zip › biomolecules-403766-SI/Supplementary Figures S1-S6.pdf]

Supplementary materials

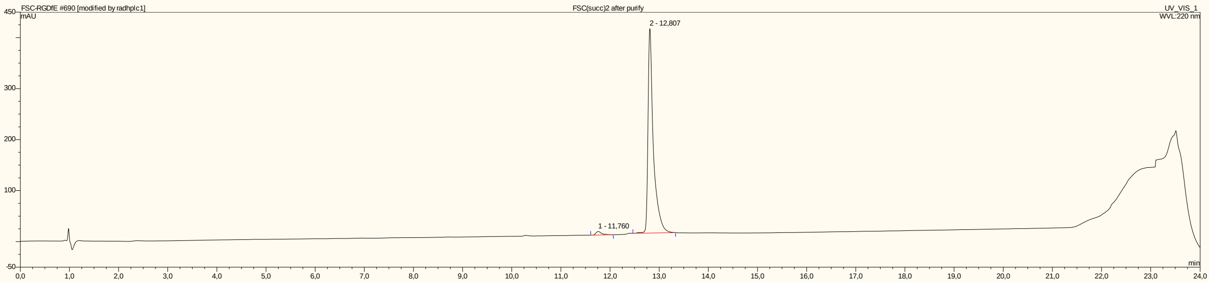

Figure 1. HPLC chromatogram of purified FSC(succ)$_2$, gradient: 0-2.0 min 0% CH_3CN/0.1% TFA, 2.0-16.0 min 0-40 % CH_3CN/0.1% TFA, t_R = 12.807 min.

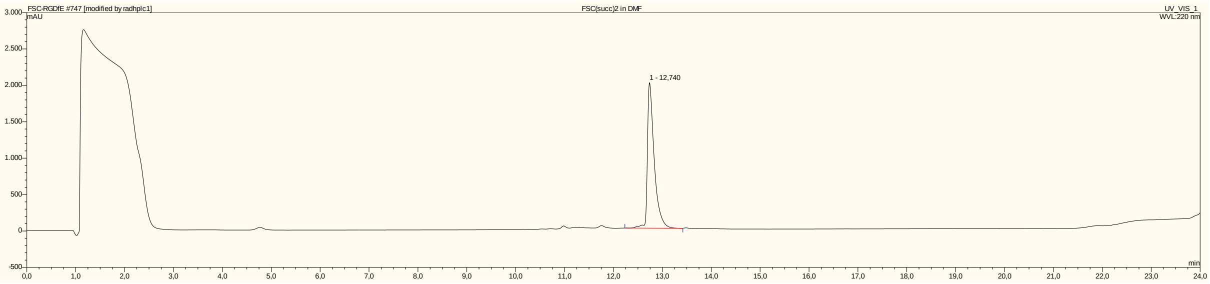

Figure S2. HPLC chromatogram of purified FSC(succ)$_2$AA, gradient: 0-2.0 min 0% CH_3CN/0.1% TFA, 2.0-16.0 min 0-40 % CH_3CN/0.1% TFA, t_R = 12.740min

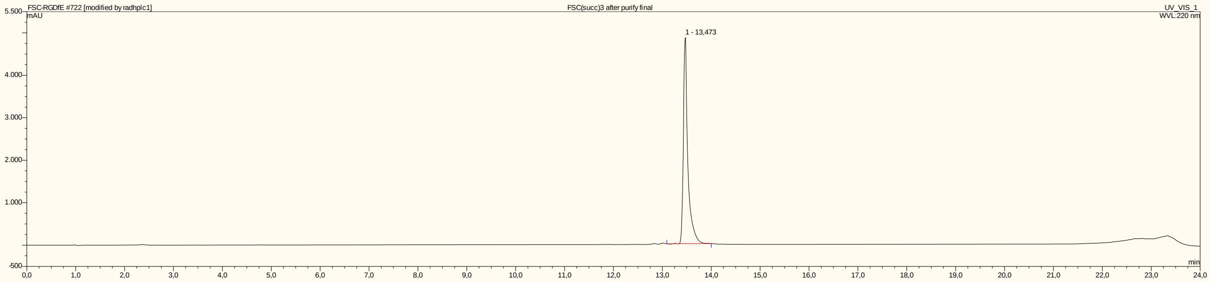

Figure S3. HPLC chromatogram of purified FSC(succ)$_3$, gradient: 0-2.0 min 0% CH_3CN/0.1% TFA, 2.0-16.0 min 0-40 % CH_3CN/0.1% TFA, t_R = 13.473 min

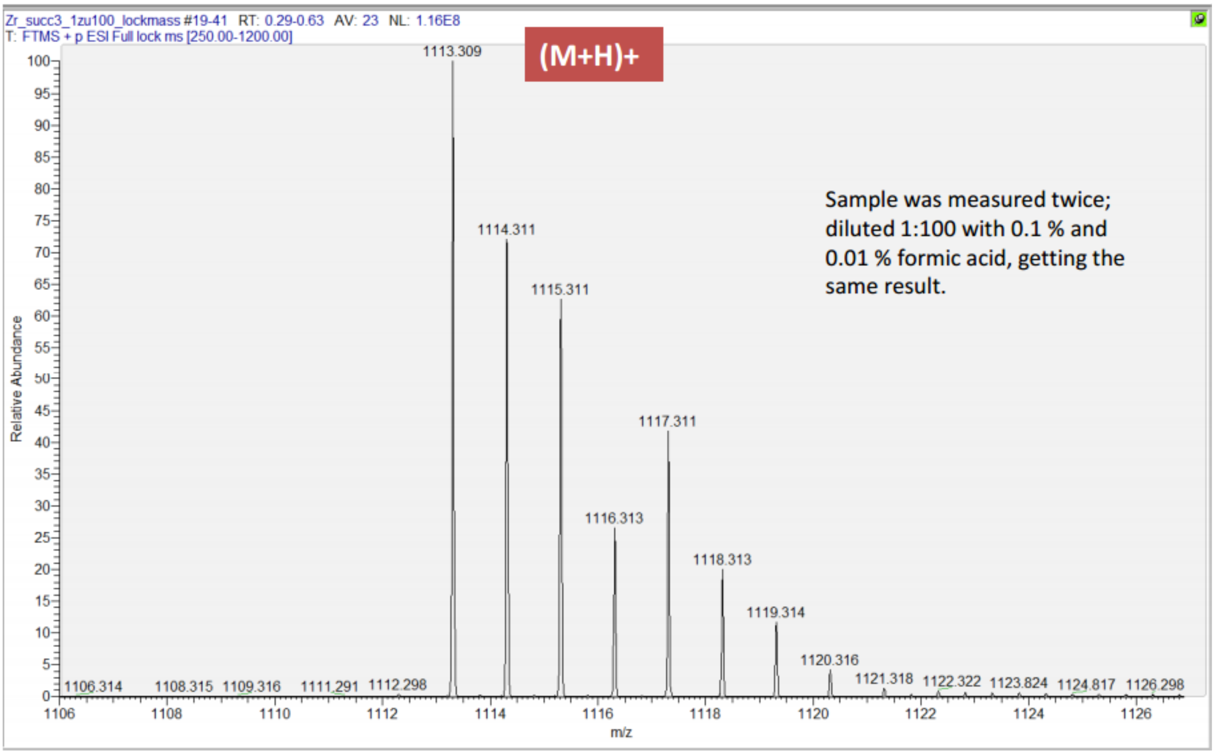

Figure S4. The ESI-MS of [natZr]FSC(succ)3

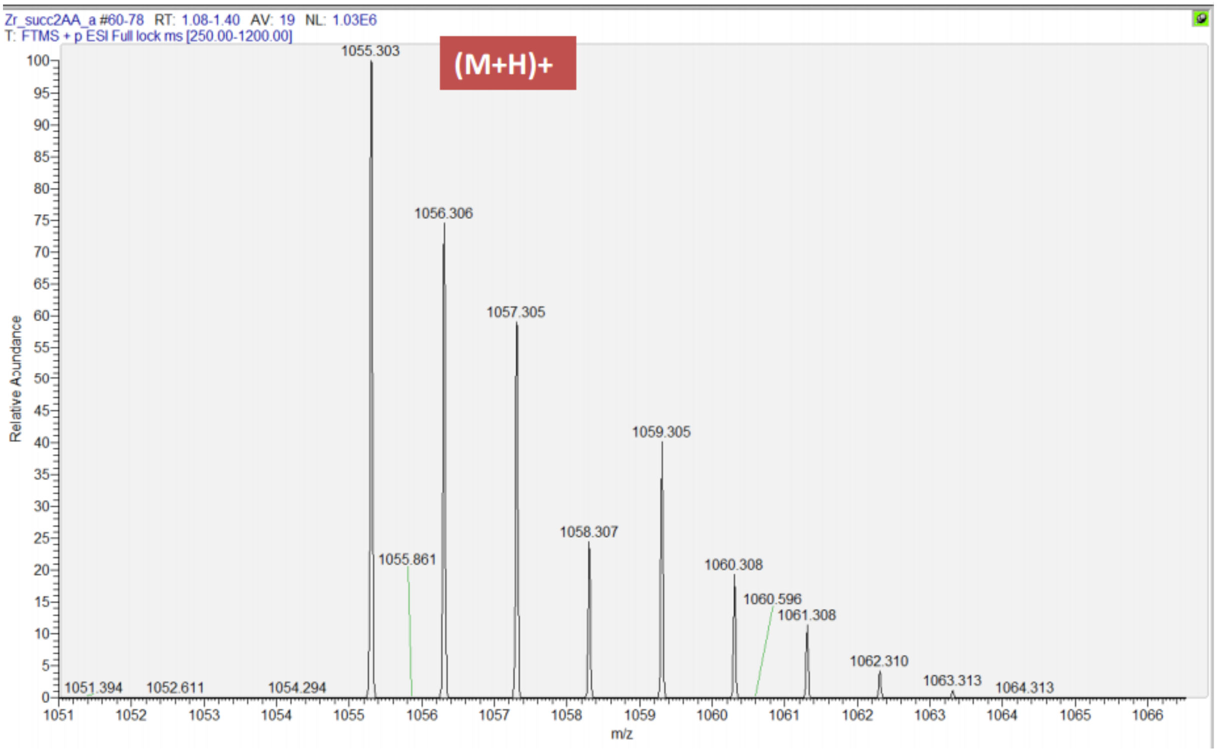

Figure S5. The ESI-MS of [natZr]FSC(succ)2AA

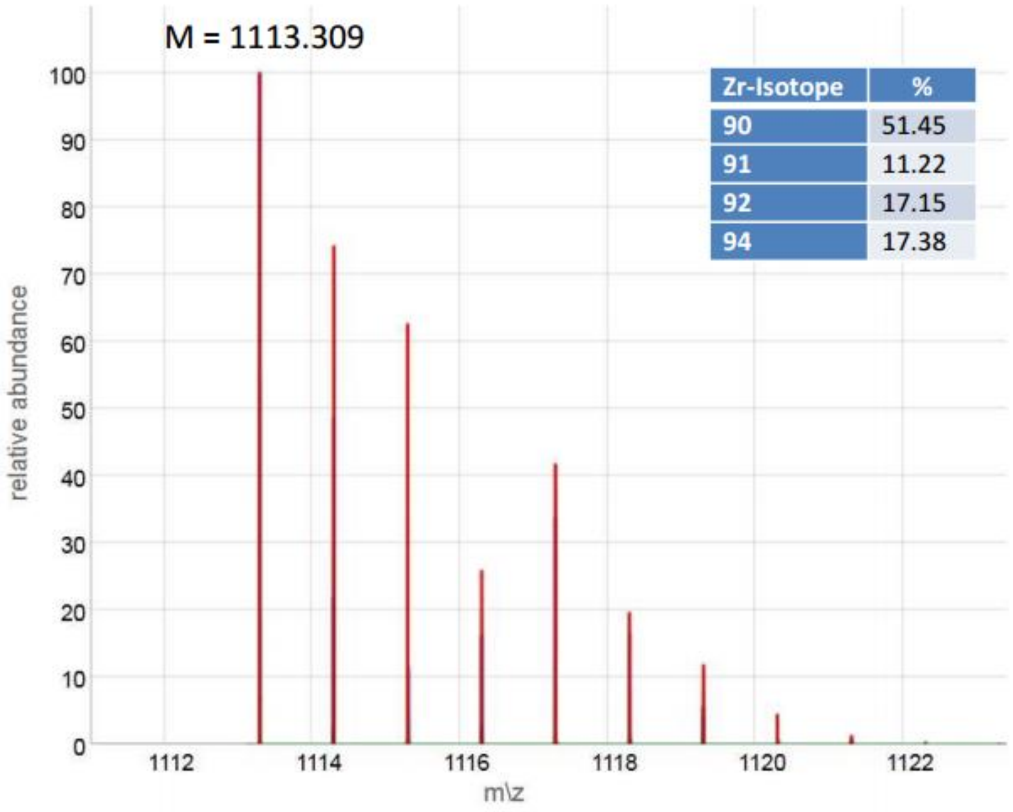

Figure S6. The calculated isotope pattern for $C_{45}H_{63}N_6O_{21}Zr$ ([^{nat}Zr]FSC(succ)$_3$)

Figure S7. 3D microPET/CT images of [^{89}Zr]Zr-FSC(succ)$_3$ in a mouse 80min p.i.
Figure S8. 3D microPET/CT images of [^{89}Zr]Zr-FSC(succ)$_3$ in a mouse 24h p.i.
Figure S9. 3D microPET/CT images of [^{89}Zr]Zr-TAFC in a mouse 80min p.i.
Figure S10. 3D microPET/CT images of [^{89}Zr]Zr-TAFC in a mouse 24h p.i.
