# Supplementary figures and images for: Rational Design, Synthesis and Preliminary Evaluation of Novel Fusarinine C-Based Chelators for Radiolabeling with Zirconium-89
Source: Biomolecules. 2019 Mar 6;9(3):91. doi: 10.3390/biom9030091 (PMC6468543; doi:10.3390/biom9030091)

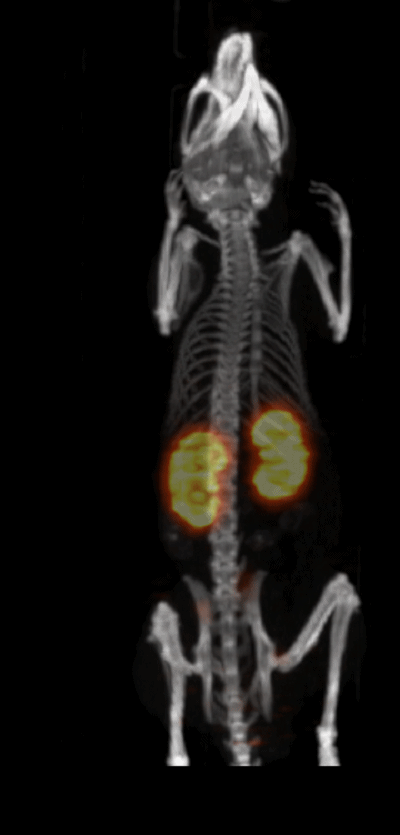

Supplement: Supplementary file 1 [file biomolecules-09-00091-s001.zip › biomolecules-403766-SI/Figure S10. 3D microPETCT images of (89Zr)Zr-TAFC in a mouse 24h p.i.gif]

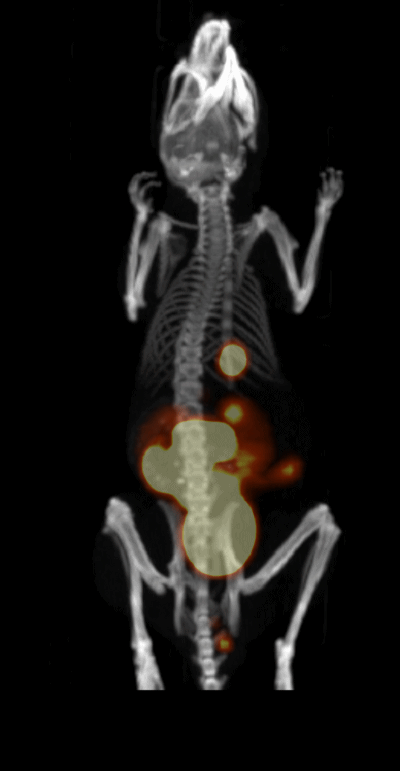

Supplement: Supplementary file 1 [file biomolecules-09-00091-s001.zip › biomolecules-403766-SI/Figure S7. 3D microPETCT images of (89Zr)Zr-FSC(succ)3 in a mouse 80min p.i.gif]

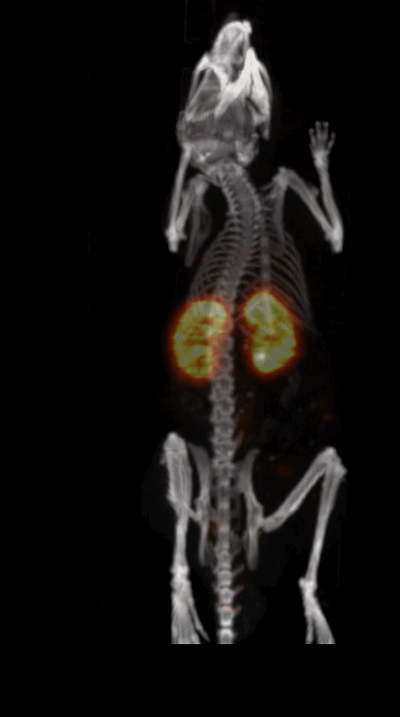

Supplement: Supplementary file 1 [file biomolecules-09-00091-s001.zip › biomolecules-403766-SI/Figure S8. 3D microPETCT images of (89Zr)Zr-FSC(succ)3 in a mouse 24h p.i.gif]

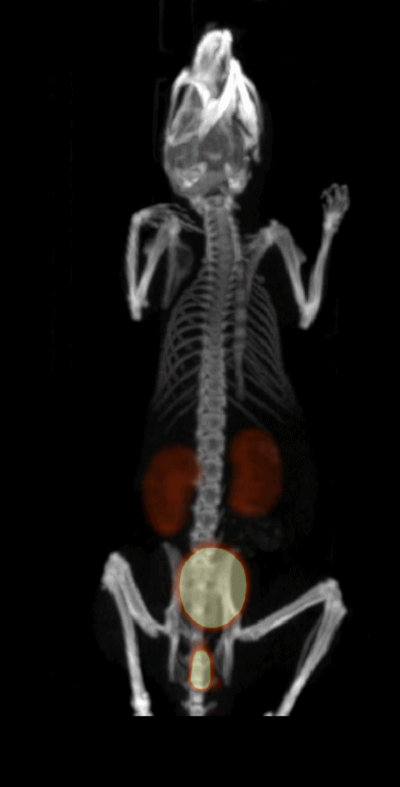

Supplement: Supplementary file 1 [file biomolecules-09-00091-s001.zip › biomolecules-403766-SI/Figure S9. 3D microPETCT images of (89Zr)Zr-TAFC in a mouse 80min p.i.gif]
